# Variable selection when estimating effects in external target populations

**DOI:** 10.1093/aje/kwae048

**Published:** 2024-04-15

**Authors:** Michael Webster-Clark, Rachael K Ross, Alexander P Keil, Robert W Platt

**Affiliations:** Department of Epidemiology, Biostatistics and Occupational Health, School of Population and Global Health, McGill University, Montreal, QC H3A 1G1, Canada; Department of Epidemiology, Gillings School of Global Public Health, University of North Carolina at Chapel Hill, Chapel Hill, NC 27599, United States; Department of Epidemiology, Gillings School of Global Public Health, University of North Carolina at Chapel Hill, Chapel Hill, NC 27599, United States; Department of Epidemiology, Mailman School of Public Health, Columbia University, New York, NY 10032, United States; National Cancer Institute, Rockville, MD 20850, United States; Department of Epidemiology, Biostatistics and Occupational Health, School of Population and Global Health, McGill University, Montreal, QC H3A 1G1, Canada

**Keywords:** external validity, generalizability, transportability, standardization, odds weights

## Abstract

External validity is an important part of epidemiologic research. To validly estimate effects in specific external target populations using a chosen effect measure (ie, “transport”), some methods require that one account for all effect measure modifiers (EMMs). However, little is known about how including other variables that are not EMMs (ie, non-EMMs) in adjustment sets affects estimates. Using simulations, we evaluated how inclusion of non-EMMs affected estimation of the transported risk difference (RD) by assessing the impacts of covariates that (1) differ (or not) between the trial and the target, (2) are associated with the outcome (or not), and (3) modify the RD (or not). We assessed variation and bias when covariates with each possible combination of these factors were used to transport RDs using outcome modeling or inverse odds weighting. Inclusion of variables that differed in distribution between the populations but were non-EMMs reduced precision, regardless of whether they were associated with the outcome. However, non-EMMs associated with selection did not amplify bias resulting from omission of necessary EMMs. Including all variables associated with the outcome may result in unnecessarily imprecise estimates when estimating treatment effects in external target populations.

## Introduction

Estimation of treatment effects in specific target populations is recognized as a core component of epidemiologic research.^1–4^ Study samples, whether in randomized trials or nonexperimental cohort studies, often differ substantially from the populations in which interventions will ultimately be applied. Clinical trials are frequently drawn from populations with a high rate of the outcome of interest and a low rate of competing risks^5^; pharmacoepidemiologic study populations are often limited based on the types of health insurance patients possess, particularly in the United States^6^^,^^7^; and some communities are reluctant to participate in scientific research for a variety of reasons.^8^ Even if study participants are randomly sampled from (or occur within the entirety of) a given target population, that sample will not be a random sample from other target populations that may be of interest to public health stakeholders.^9^

Fortunately, there are analytical approaches to account for differences between study participants and the target population. The same methods we use to estimate treatment effects for the study sample (improving internal validity) can be adapted to estimate treatment effects for other target populations (improving external validity) under parallel causal identification assumptions (ie, conditional exchangeability with positivity and causal consistency).^3^^,^^10^ These methods can generally be adapted to either generalizing (ie, estimating an effect in a population including part of the study sample)^11^ or transporting (ie, estimating an effect in a population completely external to the study sample).^12^^,^^13^

When estimating effects in the study sample and adjusting for confounding to achieve internal validity, we need to adjust for a minimally sufficient adjustment set that blocks all confounding paths from treatment to the outcome to achieve conditional exchangeability.^4^ It has been demonstrated that inclusion of variables outside these minimally sufficient adjustment sets affects precision^14^^,^^15^ and, under certain circumstances, bias.^16^ Specifically, including variables that are conditionally associated with the outcome but not treatment (ie, outcome predictors) can sometimes increase precision, while including variables that are conditionally associated with treatment but not the outcome (ie, instruments) can decrease precision and increase bias from residual confounding (ie, *bias amplification*).

When estimating a risk difference (RD) or risk ratio (RR) in an external population, we can similarly create minimally sufficient adjustment sets of effect measure modifiers (EMMs) that differ in distribution between the study sample and the target population.^17^ We might then naturally wonder whether we observe similar trends with respect to increased variance and potential bias for variables outside this adjustment set. In this case, we would be considering variables that are EMMs that do not differ in distribution between the study sample and the target population (akin to outcome predictors in the setting of internal validity) or variables that are not EMMs (ie, non-EMMs) that do differ in distribution between the study sample and the target population. Non-EMMs with paths to the outcome are of particular interest given their routine inclusion in adjustment sets that work across multiple effect measures or when using graphical approaches to identify sufficient sets for transporting or generalizing treatment effect estimates.^13^^,^^18^

In this paper, we use simulation to explore how inclusion of variables not necessary for external conditional exchangeability affects precision when using weighting or outcome modeling approaches to estimate an externally valid RD in a specific target population. We also examine whether bias amplification occurs in the presence of EMM that has not been accounted for.

## Methods

### Target parameter

We primarily focused on using a study sample $P=0$ to estimate the RD for a binary treatment *X* on a binary outcome *Y* in an external target population $P=1$ (ie, *transporting* a treatment effect). Formally, our target parameter is $\mathit{\Pr}\left({Y}^1=1|P=1\right)-\mathit{\Pr}\left({Y}^0=1|P=1\right)$, where ${Y}^x$ is the potential outcome when $X=x$. Secondarily, we examined whether our results extended to the RR and transporting effects of continuous (rather than binary) exposures.

### Defining EMMs

An EMM is a variable across which the effect of a treatment *X* on an outcome *Y* varies on a defined scale.^19^^,^^20^ For example, diabetes is an EMM for the effect of warfarin on stroke on the RD scale if the RD in persons with diabetes differs from the RD in persons without diabetes. Following definitions from previous work,^17^  $Z$ is a marginal EMM for the RD if


\begin{align*} &\mathit{\Pr}\left({Y}^1=1|Z=1\right)-\mathit{\Pr}\left({Y}^0=1|Z=1\right)\ne \mathit{\Pr}\left({Y}^1=1|Z=0\right)\\&\quad -\mathit{\Pr}\left({Y}^0=1|Z=0\right). \end{align*}

If the distribution of EMMs differs between a study sample and a target population, the treatment effect will differ between the study sample and the target population. Building on the example, if diabetes is more common in a randomized trial than in the target population, the RD estimated in the trial will not equal the RD in the target population. This type of effect measure modification is frequently identified by conventional subgroup analyses or interaction terms in linear models on the scale of interest.

EMMs can also be defined *conditionally*, rather than *marginally*.^17^ Conditional EMMs are variables across which the treatment effect varies even while fixing a specific set of variables; such a conditional EMM is useful for defining minimally sufficient sets to transport or generalize the RD or RR. Here, we focus on potential modifiers **Z** that are all independent of one another, meaning that all marginal EMMs are conditional EMMs and vice versa. In other words, there are no cases of indirect marginal EMMs or EMMs by proxy as discussed by VanderWeele and Robins.^21^

### Analytical methods for external validity

Multiple methods can be used to correct for differences in the distribution of EMMs between a study sample and a target population. Here, for simplicity, we assumed that exposure was randomized in the study sample and that the target population was external to the study. Two common approaches are weighting, where the “probability” of membership in the study sample versus the target population conditional on a set of adjustment covariates **Z** is used to construct inverse odds weights (IOWs),^2^^,^^12^ and outcome modeling or G-computation, where outcome models based on a set of adjustment covariates **Z** (stratified by treatment group) fitted in the study sample are used to predict potential outcomes in target populations.^3^ Both approaches require researchers to specify a set of covariates **Z**.

### Covariate selection

**Figure 1:**
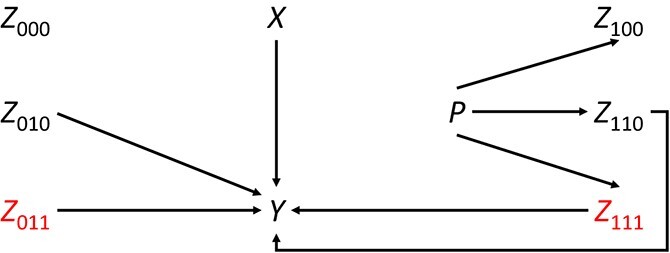
Causal relationships defining 6 types of variables when considering a randomized trial of the effect of a treatment *X* on an outcome *Y* conducted in a population *P* = 0 targeting an external population *P* = 1*.* Variables modifying the effect of *X* on *Y* are shown in red (*Z*_011_ and *Z*_111_) on the lowest line.

We examined the impact of including 6 types of independent binary variables in the adjustment sets. The types are defined by (1) whether the variable differs in distribution between the study sample and the target population, (2) whether the variable is associated with the outcome, and (3) if it is associated with the outcome, whether it is an EMM. Figure 1 is a directed acyclic graph^22^ showing the causal relationships for each type of variable. Variables shown in red on the lowest line (*Z*_011_ and *Z*_111_) modify the effect of *X* on *Y*. The covariates **Z** are indexed as *Z*_ABC_, where A, B, and C are the factors noted above and are equal to 1 or 0. *Z*_000_ is distributed identically in *P* = 0 and *P* = 1 and has no association with *Y*; *Z*_010_ is distributed identically in *P* = 0 and *P* = 1, has an effect on *Y*, but does not modify the effect of *X* on *Y* on the RD scale; *Z*_011_ is distributed identically in *P* = 0 and *P* = 1, has an effect on *Y*, and modifies the effect of *X* on *Y* on the RD scale; *Z*_100_ differs in distribution between *P* = 0 and *P* = 1 but has no association with *Y*; *Z*_110_ differs in distribution between *P* = 0 and *P* = 1 and has an effect on *Y*, but does not modify the effect of *X* on *Y* on the RD scale; and *Z*_111_ differs in distribution between *P* = 0 and *P* = 1*,* has an effect on *Y*, and modifies the effect of *X* on *Y* on the RD scale. Accounting for *Z*_111_ is minimally sufficient to transport the treatment effect from *P* = 0 and *P* = 1.

### Primary simulation

We simulated 20 000 replicates of a 10 000-person randomized trial (to avoid issues of small sample size) of the effect of a treatment *X* and an outcome *Y* in a population *P* = 0 aiming to estimate the RD in a completely external 10 000-person target population *P* = 1 in the presence of 6 binary baseline covariates corresponding to the 6 types discussed above. The probability Pr(*Y* = 1*|X,*  **Z***)* was based on a linear probability model with main-effect terms for *X*, *Z*_010_, *Z*_011_, *Z*_110_, and *Z*_111_ and interaction terms for interaction of $X$ with *Z*_011_ and with *Z*_111_*.*  Table 1 includes the full parameterization of the simulation; SAS code for recreating the simulation can be found online (https://github.com/mawcpharmdphd/ext_var_sel).

**Table 1:** Parameters used to generate simulated data and explore the impact of various adjustment sets on the bias and precision of transported effect estimates.

**Parameter**	**Trial value**	**Target value**
No. of simulation replicates	10 000	10 000
Trial population size, *n*	10 000	10 000
Probability of *Z*_000_^a^	0.5	0.5
Probability of *Z*_010_	0.5	0.5
Probability of Z_011_	0.5	0.5
Probability of Z_100_	0.2	0.8
Probability of Z_110_	0.2	0.8
Probability of *Z*_111_	0.2	0.8
Probability of *Y*	$0.1+0.1\times X+0.1\times Z2+0.1\times Z3+0.1\times Z5+0.1\times Z6+0.1\times X\times Z3+0.1\times X\times Z6$	

^a^“*Z*_abc_” indicates whether a variable is independent of *P* (a), whether it is independent of *Y* (b), and whether it is an effect measure modifier (c).

### Analyses

We transported the RD from *P* = 0 to *P* = 1 using 12 possible adjustment sets with no interaction terms. For the IOW approach, adjustment sets were included in a linear regression model predicting the probability of sampling; for the outcome modeling approach, these sets were included in a treatment-stratified, linear outcome model. We left the first set empty to assess the crude results. The next set included only *Z*_111_, the only EMM that differs between *P* = 0 and *P* = 1, which thus comprised a minimally sufficient set to transport the treatment effect between the 2 populations. The next 5 sets sequentially added each of the other covariates individually to this minimally sufficient model (eg, so that each set included *Z*_111_ and 1 other variable). Finally, the last 5 sets included *Z*_000_*, Z*_010_, *Z*_011_, *Z*_100_*,* or *Z*_110_  *without Z*_111_ to assess the potential for *bias amplification*. Table 2 summarizes the questions answered by each of the 11 models.

**Table 2:** Questions answered by each of the 11 adjustment sets examined in the main analysis.

**Adjustment set**	**Minimally sufficient**	**“Sampling” association of non-*Z***_**111**_ **covariate**	**Outcome association of non-*Z***_**111**_ **covariate**	**Non-*Z***_**111**_ **covariate is an EMM**	**Central focus**
Empty set					Crude results
*Z*_111_ alone	X				SE
*Z*_000_ + *Z*_111_	X				SE
*Z*_010_ *+ Z*_111_	X		X		SE
*Z*_011_ *+ Z*_111_	X		X	X	SE
*Z*_100_ *+ Z*_111_	X	X			SE
*Z*_110_ *+ Z*_111_	X	X	X		SE
*Z*_000_ alone					Bias
*Z*_010_ alone			X		Bias
*Z*_011_ alone			X	X	Bias
*Z*_100_ alone		X			Bias
*Z*_110_ alone		X	X		Bias

Abbreviation: EMM, effect measure modifier.

### Alternative scenarios and sensitivity analyses

We explored several additional scenarios and sensitivity analyses. First, we repeated the analyses with a 1000-person trial and a 10 000-person target population or a 10 000-person trial and a 100 000-person target population, rather than equal-sized populations, to explore whether relative variance increases changed. Next, we reduced the difference between *P* = 0 and *P* = 1 with respect to *Z*_100_*, Z*_110_*,* and *Z*_111_*.* We also assessed whether similar results were observed for the RR both in the original scenario and when we introduced a new variable associated with *Y* and *Z*_111_ that differed in distribution between *P* = 0 and *P* = 1 (with a direct association with *P* in addition to its relationship with *Z*_111_) and expanded the minimally sufficient adjustment set. We also examined whether similar results were observed when *X* or *Z* was a continuous standard normal (rather than a binary) variable.

## Results

### Simulation results

Table 3 shows mean transported RDs and empirical SEs calculated across all 20 000 simulation replicates.

**Table 3:** Mean transported RDs and their empirical SEs across 20 000 simulation replicates for each adjustment set.

		**Model**			
		**Inverse odds weighting**		**Outcome**	
**Adjustment set**	**Mean transported RD estimate**	**Empirical SE of the RD**	**Relative increase in SE**	**Empirical SE of the RD**	**Relative increase in SE**
Empty set	0.170	0.0093	Referent for “alone”	0.0093	Referent for “alone”
*Z*_111_ alone	0.230	0.0174	Referent for “+Z_111_”	0.0176	Referent for “+Z_111_”
*Z*_000_ + *Z*_111_	0.230	0.0174	1.000	0.0176	1.001
*Z*_010_ *+ Z*_111_	0.230	0.0174	1.000	0.0175	0.995
*Z*_011_ *+ Z*_111_	0.230	0.0174	1.000	0.0173	0.983
*Z*_100_ *+ Z*_111_	0.230	0.0313	1.812	0.0224	1.271
*Z*_110_ *+ Z*_111_	0.230	0.0317	1.824	0.0226	1.285
*Z*_000_ alone	0.170	0.0092	1.000	0.0097	1.018
*Z*_010_ alone	0.170	0.0092	1.000	0.0096	1.006
*Z*_011_ alone	0.170	0.0092	1.000	0.0096	1.003
*Z*_100_ alone	0.170	0.0166	1.804	0.0170	1.772
*Z*_110_ alone	0.170	0.0173	1.877	0.0177	1.844

Abbreviation: RD, risk difference.

### IOW findings

As expected, every adjustment set including *Z*_111_ (the minimally sufficient adjustment set) yielded an unbiased RD in the target population of 0.230. Using *Z*_111_ alone resulted in an empirical SE of 0.0174. Including variables that were identically distributed in *P* = 0 and *P* = 1 (*Z*_000_, *Z*_010_, and *Z*_011_) in the adjustment set alongside *Z*_111_ did not change precision, even if they were associated with the outcome or an EMM for the RD (this may not hold for smaller samples due to cells that are empty by chance). On the other hand, including variables that differed between *P* = 0 and *P* = 1 in adjustment sets that were not EMMs alongside *Z*_111_ substantially decreased precision, whether these variables were independent of the outcome (*Z*_100_ empirical SE = 0.0313, an 80% increase in SE) or associated with the outcome (*Z*_110_ empirical SE = 0.0317, an 82% increase).

When *Z*_111_ was omitted from the adjustment set, all adjustment sets yielded biased RD estimates in the target population of 0.170 (identical to the crude). The adjustment sets including only *Z*_100_ and *Z*_110_ were once again the least precise (*Z*_100_ SE, 0.0166; *Z*_110_ SE, 0.0173). There was no evidence of amplification of the bias resulting from omitting *Z*_111_ in the adjustment set when adjusting for any of the various *Z*’s.

### Outcome modeling findings

Mean RD estimates were identical to the IOW results. Including Z_010_ and Z_011_ (outcome predictors that did not differ between populations) reduced the precision of the transported RD. There was again less precision when *Z*_100_ and *Z*_110_ were included, but the loss of precision was smaller than that observed when using IOWs (*Z*_100_ relative increase in SE = 27.1%; *Z*_110_ relative increase in SE = 28.5%). The loss of precision when *Z*_111_ was omitted was about the same as with IOWs, however. There was still no evidence of bias amplification.

### Sensitivity analyses

Table 4 shows the results of each sensitivity analysis, with Tables S1–S7 including full results for each. Results were generally consistent with the base case. When the *P* = 0 population was much smaller than the *P* = 1 population (1000-person trial and 10 000-person target or 10 000-person trial and 100 000-person target), we observed larger relative increases in SE and small deviations from the true RD of 0.230 when including *Z*_110_ and *Z*_100_. As one might expect, the precision loss from including *Z*_110_ and *Z*_100_ was reduced when their distributions were more similar between *P* = 1 and *P* = 0. On the other hand, the increase in the empirical SE of the log RR was larger than the increase in the SE of the RD, both with and without the addition of another outcome predictor that was associated with *Z*_111_ differently between the two populations. Using a continuous *X* or 3 continuous *Z*’s did not result in any changes in the findings. Most interestingly, across *all* sensitivity analyses, adjusting for *Z*_110_ (the non-EMM related to the outcome that differed between study and target) resulted in a greater loss of precision than adjusting for *Z*_100_ (the non-EMM independent of the outcome that differed between study and target), with this difference being more pronounced in some sensitivity analysis than in others, suggesting that the larger increase with *Z*_110_ in the base case was not a chance finding due to simulation error.

**Table 4:** Sensitivity analysis results.

**Analysis**	**Motivation**	**Findings**
Smaller *P* = 0 (trial) population than the base case (1000 people)	Explore whether our findings changed with a smaller trial size.	Consistent with overall findings, but with a larger relative variance increase and small deviations from the true RD of 0.230.
Larger *P* = 1 (target) population (100 000 people) than the base case	Explore whether our findings changed with a larger target population size.	Consistent with overall findings, but with a larger relative variance increase and small deviations from the true RD of 0.230.
Reduced covariate difference between *P* = 0 and *P* = 1	Explore whether these findings only occurred with extreme covariate gaps.	Variance still increased, but the increase was less than in the base case.
RR scenario with no additional covariates	Explore the degree of variance increase for the RR, rather than the RD.	Variance increased even more than in the base case; variance increase was much more pronounced with *Z*_110_ than with *Z*_100_.
RR scenario with a new covariate associated with the EMM *Z*_111_	Explore whether the RR results changed after introducing a new indirect EMM associated with the outcome.	Variance increased similarly to the RR scenario; slight bias when the new covariate was omitted.
Continuous *X*	Explore whether results were similar when transporting the effects of a continuous *X*, rather than a binary *X.*	Similar variance increase to the base case; variance increase was more pronounced when including *Z*_110_ than with *Z*_100_.
Continuous *Z*	Explore whether results were similar when transporting a binary *X* in the presence of a continuous *Z*, rather than a binary *Z.*	Variance increase as in the base case, with a more pronounced variance increase from including *Z*_110_ than with *Z*_100_.

Abbreviations: EMM, effect measure modifier; RD, risk difference; RR, risk ratio.

## Discussion

Our results suggest that, when estimating the RR or RD, adjusting for independent non-EMMs that differ between study and target populations using weighting or outcome modeling decreases precision, regardless of whether those variables are associated with the outcome. Adjusting for non-EMMs does not, however, amplify bias resulting from the omission of important EMMs from the adjustment set. When trying to maximize the precision of an RD or RR estimate transported to an external target population, researchers should prioritize the inclusion of EMMs (regardless of whether they differ between the study and target populations) and try to avoid including non-EMMs that differ between the study and target populations (regardless of whether they are associated with the outcome).

These results are somewhat surprising given the findings of studies exploring variable selection in internal validity problems. After all, identical analytical solutions (weighting and outcome modeling) are being used to account for threats to both internal and external validity. Intuition would suggest that including outcome predictors in our weighting or outcome models would increase efficiency, regardless of whether they are EMMs, and that including unnecessary variables that differ between study and target would amplify bias from EMMs omitted from our adjustment models. These two intuitions fail for 2 different, equally important, reasons.

### Why the intuitions fail

#### Reason #1: EMMs play the role in external validity that outcome predictors play in internal validity

This is the reason that our simulated findings observe losses of precision when outcome predictors are included in adjustment sets, rather than the gains observed in internal validity. If we want to extend our intuitions from internal validity to external validity, we need to take into account that the equivalent of “outcome predictors” when transporting treatment effects is an EMM for the treatment effect of interest. While estimation of an internally valid treatment effect requires conditional exchangeability between treatment arms with respect to the outcome, estimation of an externally valid treatment effect requires conditional exchangeability between study populations with respect to EMMs.^1^ While all EMMs are associated with the outcome independently of treatment, not all variables associated with the outcome will act as EMMs on a given scale. The variables analogous to “instruments of exposure” are variables that are associated with “sampling” into the study that are not EMMs, *regardless* of whether they are associated with the outcome.

Inclusion of these variables in the adjustment set results in unnecessarily extreme weights (for IOWs) or estimating coefficients in sparser spaces (for outcome modeling methods) with no gains in terms of the treatment effect estimates. In other words, the closest analogue to “adjust for all predictors of the outcome as potential confounders and avoid instruments” in the setting of external validity is “adjust for all EMMs on the scale of interest and avoid non-EMMs that are distributed differently between the trial and the target.”

#### Reason #2: the direction of causal arrows related to external target populations differs from the direction of causal arrows in internal validity

This is why we do not observe the bias amplification from the non-EMM that instruments create in an internal validity setting. Instruments amplify bias when included in adjustment sets because they are associated with unmeasured confounding variables within strata of the exposed and unexposed; by conditioning on them, you increase the amount of variation explained by the confounder. This forces the exposed and unexposed farther apart when instruments are included in adjustment sets (for a detailed mathematical description, see Bhattacharya and Vogt,^23^ Pearl,^16^ and Myers et al^24^).

On the other hand, the way we simulated our study and target population data is most compatible with treating the covariates as consequences of sampling into the study (ie, arrows flowing out of the study population into covariates). As a result, none of the variation in the unmeasured EMM is explained by the measured non-EMM in the way that the variation in unmeasured confounders is explained by measured instruments. When we simulated analogous situations in the setting of internal validity with similar independences within treatment strata, we did not observe any bias amplification.

### Limitations

#### Simulation limitations

Our primary simulation focused on independent and binary covariates (with the exception of the RR and sensitivity analyses) to avoid having to deal with additional complexities that arise when variables may be conditionally associated but marginally unassociated with the outcome and vice versa. Further nuance, like collider bias, may be introduced when variables are associated with one another or exhibit joint interactions with the outcome. Our main simulation also used fairly large trial and target populations of similar sizes, which may not be representative of real-world applications of these methods; that being said, a smaller trial and larger target were associated with larger relative loss of precision and minute added bias when adjusting for unnecessary variables. We also focused on simple binary outcomes and the RD and RR, the two simplest effect measures with derivable minimally sufficient adjustment sets. Studying more complex cases and more complex effect measures will be key to expanding and verifying these or similar heuristics in new and different contexts.

#### Additional limitations

We focused on *effects* rather than *risks* or *measures of occurrence*. If researchers are interested in estimating population measures of event occurrence (eg, risks, rates, or survival times) under *X* = 1 and *X* = 0, the adjustment set will generally require outcome predictors regardless of whether they are EMMs for any or all effect measures of interest.^13^ While non-EMM outcome predictors that differ between populations will similarly decrease precision when adjusted for, they are still necessary for unbiased estimators of risks and measures of occurrence. Finally and perhaps most importantly, identifying whether variables are EMMs in real data is not easy, even if researchers are studying the RD. Conventional methods that distinguish EMMs from non-EMMs tend to perform quite poorly.^20^^,^^25^ Moreover, which types of EMMs (marginal or conditional) researchers need to identify varies by effect measure.^17^ More work needs to be done to understand how to maximize the efficiency of these methods and create the kinds of heuristics necessary to perform the sorts of algorithmic variable selection common in machine learning methods. Until then, focusing on variables that are *potential* EMMs (which, as a general rule, consists of variables associated with the outcome)^18^ can minimize the risk of bias at the expense of precision. Ultimately, researchers may face a similar bias-variance trade-off to the one present when deciding adjustment sets for internal validity.

### Conclusion

Variable selection when transporting or generalizing treatment effects to specific populations involves considering whether variables differ between the study and target populations, as well as whether those variables modify treatment effects of interest. The ideal adjustment set for external validity includes EMMs that differ between the trial and target populations. Casually extending heuristics built within the context of internal validity, like including all outcome predictors to improve precision, to the setting of external validity can lead to incorrect conclusions.

## Supplementary Material

Web_Material_kwae048

## Data Availability

Simulation and analytical software code is available upon request.

## References

[1] Westreich D, Edwards JK, Lesko CR, et al. Target validity and the hierarchy of study designs. *Am J Epidemiol*. 2019;188(2):438–443. 10.1093/aje/kwy228 30299451 PMC6357801

[2] Dahabreh IJ, Haneuse SJA, Robins JM, et al. Study designs for extending causal inferences from a randomized trial to a target population. *Am J Epidemiol*. 2021;190(8):1632–1642. 10.1093/aje/kwaa270 33324969 PMC8536837

[3] Hernán MA, VanderWeele TJ. Compound treatments and transportability of causal inference. *Epidemiology*. 2011;22(3):368–377. 10.1097/EDE.0b013e3182109296 21399502 PMC3805254

[4] Rothman KJ, Greenland S, Lash TL. Modern Epidemiology. 3rd ed. Lippincott Williams & Wilkins; 2008.

[5] Talarico L, Chen G, Pazdur R. Enrollment of elderly patients in clinical trials for cancer drug registration: a 7-year experience by the US Food and Drug Administration. *J Clin Oncol*. 2004;22(22):4626–4631. 10.1200/JCO.2004.02.175 15542812

[6] Mitchell JB, Bubolz T, Paul JE, et al. Using Medicare claims for outcomes research. *Med Care*. 1994;32(7 suppl):JS38–JS51. 10.1097/00005650-199407001-00004 8028412

[7] Kulaylat AS, Schaefer EW, Messaris E, et al. Truven Health Analytics MarketScan databases for clinical research in colon and rectal surgery. *Clin Colon Rectal Surg*. 2019;32(1):54–60. 10.1055/s-0038-1673354 30647546 PMC6327721

[8] Hamel LM, Penner LA, Albrecht TL, et al. Barriers to clinical trial enrollment in racial and ethnic minority patients with cancer. *Cancer Control*. 2016;23(4):327–337. 10.1177/107327481602300404. 27842322 PMC5131730

[9] Rothman KJ, Gallacher JE, Hatch EE. Why representativeness should be avoided. *Int J Epidemiol*. 2013;42(4):1012–1014. 10.1093/ije/dys223 24062287 PMC3888189

[10] Dahabreh IJ, Robertson SE, Steingrimsson JA, et al. Extending inferences from a randomized trial to a new target population. *Stat Med*. 2020;39(14):1999–2014. 10.1002/sim.8426 32253789

[11] Cole SR, Stuart EA. Generalizing evidence from randomized clinical trials to target populations: the ACTG 320 trial. *Am J Epidemiol*. 2010;172(1):107–115. 10.1093/aje/kwq084 20547574 PMC2915476

[12] Westreich D, Edwards JK, Lesko CR, et al. Transportability of trial results using inverse odds of sampling weights. *Am J Epidemiol*. 2017;186(8):1010–1014. 10.1093/aje/kwx164 28535275 PMC5860052

[13] Pearl J, Bareinboim E. External validity: from do-calculus to transportability across populations. *Stat Sci*. 2014;29(4):579–595. 10.1214/14-STS486

[14] Brookhart MA, Schneeweiss S, Rothman KJ, et al. Variable selection for propensity score models. *Am J Epidemiol*. 2006;163(12):1149–1156. 10.1093/aje/kwj149 16624967 PMC1513192

[15] Robinson LD, Jewell NP. Some surprising results about covariate adjustment in logistic regression models. *Int Stat Rev*. 1991;59(2):227–240. 10.2307/1403444

[16] Pearl J . Invited commentary: understanding bias amplification. *Am J Epidemiol*. 2011;174(11):1223–1227. 10.1093/aje/kwr352 22034488 PMC3224255

[17] Webster-Clark M, Keil AP. How effect measure choice influences minimally sufficient adjustment sets for external validity. *Am J Epidemiol*. 2023;192(7):1148–1154. 10.1093/aje/kwad041 36813295

[18] Webster-Clark M, Breskin A. Directed acyclic graphs, effect measure modification, and generalizability. *Am J Epidemiol*. 2021;190(2):322–327. 10.1093/aje/kwaa185 32840557

[19] VanderWeele TJ . On the distinction between interaction and effect modification. *Epidemiology*. 2009;20(6):863–871. 10.1097/EDE.0b013e3181ba333c 19806059

[20] VanderWeele TJ . Confounding and effect modification: distribution and measure. *Epidemiol Methods*. 2012;1(1):55–82. 10.1515/2161-962X.1004 25473593 PMC4249691

[21] VanderWeele TJ, Robins JM. Four types of effect modification: a classification based on directed acyclic graphs. *Epidemiology*. 2007;18(5):561–568. 10.1097/EDE.0b013e318127181b 17700242

[22] Pearl J . Causal diagrams for empirical research. *Biometrika*. 1995;82(4):669–688. 10.1093/biomet/82.4.669

[23] Bhattacharya J, Vogt WB. Do Instrumental Variables Belong in Propensity Scores? National Bureau of Economic Research; 2007.

[24] Myers JA, Rassen JA, Gagne JJ, et al. Effects of adjusting for instrumental variables on bias and precision of effect estimates. *Am J Epidemiol*. 2011;174(11):1213–1222. 10.1093/aje/kwr364 22025356 PMC3254160

[25] Webster-Clark M, Baron JA, Funk MJ, et al. How subgroup analyses can miss the trees for the forest plots: a simulation study. *J Clin Epidemiol*. 2020;126:65–70. 10.1016/j.jclinepi.2020.06.020 32565216 PMC7529905

