# Supplementary material for: Variable selection when estimating effects in external target populations
Source: Am J Epidemiol. 2024 Apr 15;193(8):1176–81. doi: 10.1093/aje/kwae048 (PMC11299018; doi:10.1093/aje/kwae048)
Supplement: Web_Material_kwae048 [file web_material_kwae048.pdf]

Supplementary Material

Variable Selection When Estimating Effects in External Target Populations

Michael Webster-Clark, Rachael K. Ross, Alexander P. Keil, and Robert W. Platt

Table of Contents

Table S1: Smaller trial results____________________________________Page 2

Table S2: Larger target results____________________________________Page 3

Table S3: Decreased difference results____________________________Page 4

Table S4: Risk ratio results_______________________________________Page 5

Table S5: Risk ratio additional Z results___________________________Page 6

Table S6: Continuous X results___________________________________Page 7

Table S7: Continuous Z results___________________________________Page 8

Table S1: Smaller trial results

**Adjustment set**	**Risk difference**	**Standard error**
Empty set	0.170	0.0292
Z111 alone	0.230	0.0552
Z000+Z111	0.230	0.0553
Z010+Z111	0.230	0.0554
Z011+Z111	0.230	0.0553
Z100+Z111	0.232	0.1163
Z110+Z111	0.232	0.1188
Z000 alone	0.170	0.0292
Z010 alone	0.170	0.0292
Z011 alone	0.170	0.0291
Z100 alone	0.170	0.0528
Z110 alone	0.170	0.0557

Table S2: Larger target results

Adjustment set	Risk difference	Standard error
Empty set	0.170	0.0093
Z111 alone	0.230	0.0174
Z000+Z111	0.230	0.0174
Z010+Z111	0.230	0.0174
Z011+Z111	0.230	0.0365
Z100+Z111	0.232	0.0367
Z110+Z111	0.232	0.0093
Z000 alone	0.170	0.0093
Z010 alone	0.170	0.0093
Z011 alone	0.170	0.0092
Z100 alone	0.170	0.0167
Z110 alone	0.170	0.0174

Table S3: Decreased difference results

Adjustment set	Risk difference	Standard error
Empty set	0.190	0.0095
Z111 alone	0.210	0.0105
Z000+Z111	0.210	0.0105
Z010+Z111	0.210	0.0105
Z011+Z111	0.210	0.0105
Z100+Z111	0.210	0.0113
Z110+Z111	0.210	0.0115
Z000 alone	0.190	0.0096
Z010 alone	0.190	0.0096
Z011 alone	0.190	0.0096
Z100 alone	0.190	0.0104
Z110 alone	0.190	0.0106

Table S4: Risk ratio results

Adjustment set	Risk difference	Log(Standard error)
Empty set	0.554	0.0250
Z111 alone	0.441	0.0569
Z000+Z111	0.441	0.0569
Z010+Z111	0.441	0.0569
Z011+Z111	0.441	0.0569
Z100+Z111	0.441	0.1034
Z110+Z111	0.440	0.1154
Z000 alone	0.554	0.0250
Z010 alone	0.554	0.0250
Z011 alone	0.554	0.0250
Z100 alone	0.554	0.0454
Z110 alone	0.554	0.0531

Table S5: Risk ratio additional Z results

**Adjustment set**	**Risk ratio**	**Log(standard error)**
Empty set	0.451	0.0635
Z111 alone	0.451	0.0635
Z000+Z111	0.451	0.0635
Z010+Z111	0.451	0.0635
Z011+Z111	0.450	0.1144
Z100+Z111	0.449	0.1283
Z110+Z111	0.559	0.0268
Z000 alone	0.559	0.0268
Z010 alone	0.559	0.0268
Z011 alone	0.559	0.0479
Z100 alone	0.559	0.0563
Z110 alone	0.559	0.0268
Z000+Z111+Z7	0.446	0.0727
Z010+Z111+Z7	0.446	0.0726
Z011+Z111+Z7	0.446	0.0726
Z100+Z111+Z7	0.444	0.1422
Z110+Z111+Z7	0.443	0.1610

Table S6: Continuous X results

Adjustment set	Risk difference	Standard error
Empty set	0.085	0.0093
Z111 alone	0.115	0.0172
Z000+Z111	0.115	0.0172
Z010+Z111	0.115	0.0172
Z011+Z111	0.115	0.0172
Z100+Z111	0.114	0.0310
Z110+Z111	0.115	0.0319
Z000 alone	0.085	0.0093
Z010 alone	0.085	0.0093
Z011 alone	0.085	0.0093
Z100 alone	0.085	0.0168
Z110 alone	0.085	0.0176

Table S7: Continuous Z results

Adjustment set	Risk difference	Standard error
Empty set	0.0376	0.0086
Z111 alone	0.0502	0.0148
Z000+Z111	0.0502	0.0148
Z010+Z111	0.0502	0.0148
Z011+Z111	0.0502	0.0148
Z100+Z111	0.0501	0.0244
Z110+Z111	0.0502	0.025
Z000 alone	0.0376	0.0086
Z010 alone	0.0376	0.0086
Z011 alone	0.0376	0.0086
Z100 alone	0.0375	0.0141
Z110 alone	0.0376	0.014
